# Notochordal and nucleus pulposus marker expression is maintained by sub-populations of adult human nucleus pulposus cells through aging and degeneration

**DOI:** 10.1038/s41598-017-01567-w

**Published:** 2017-05-04

**Authors:** Stephen M. Richardson, Francesca E. Ludwinski, Kanna K. Gnanalingham, Ross A. Atkinson, Anthony J. Freemont, Judith A. Hoyland

**Affiliations:** 1 Division of Cell Matrix Biology and Regenerative Medicine, School of Biological Sciences, Faculty of Biology, Medicine and Health, The University of Manchester, Stopford Building, Oxford Road, Manchester, M13 9PT United Kingdom; 2 Department of Neurosurgery, Salford Royal NHS Foundation Trust, Manchester Academic Health Science Centre, Salford, United Kingdom; 3 NIHR Manchester Musculoskeletal Biomedical Research Unit, Manchester Academic Health Science Centre, Central Manchester NHS Foundation Trust, Manchester, United Kingdom

## Abstract

The nucleus pulposus (NP) of the intervertebral disc (IVD) demonstrates substantial changes in cell and matrix composition with both ageing and degeneration. While recent transcriptomic profiling studies have helped define human NP cell phenotype, it remains unclear how expression of these markers is influenced by ageing or degeneration. Furthermore, cells of the NP are thought to derive from the notochord, although adult NP lacks identifiable notochordal (NC) cells. This study aimed to confirm expression of previously identified NP and NC marker genes in adult human NP cells from a range of ages and degenerate states. Importantly, using gene expression analysis (N = 60) and immunohistochemistry (N = 56) the study demonstrates expression of NP markers FoxF1, Pax-1, keratin-8/18, carbonic anhydrase-12, and NC markers brachyury, galectin-3 and CD24 in cells of the NP irrespective of age or degeneration. Our immunohistochemical data, combined with flow cytometry (N = 5) which identified a small number of CA12^+^Gal3^+^T^+^CD24^+^ cells, suggests the possible presence of a sub-population of cells with an NC-like phenotype in adult NP tissue. These findings suggest that the NP contains a heterogeneous population of cells, which may possess varied phenotypic and functional profiles and thus warrant further investigation to improve our understanding of IVD homeostasis and repair.

## Introduction

Approximately 70% of individuals in developed societies suffer from low back pain (LBP) and neck pain at some point^1, 2^. The socioeconomic impact of LBP amounts to over £12 billion in the UK alone^3^, and whilst the underlying pathologies of these are multifactorial, degeneration of the lumbar and cervical intervertebral discs (IVDs) have been directly correlated to the development of these conditions^4, 5^. Degeneration of the IVD is a progressive age-related disorder. Symptomatic relief may be achieved utilising current therapeutic strategies; however such strategies fail to address the underlying pathogenesis and aberrant cell biology and thus are ineffective for long-term treatment of this disease. The research community therefore continues to strive to improve understanding of the cellular and molecular biology of the healthy and degenerate disc in order to inform development of novel regenerative strategies.

In order for effective regenerative strategies to be developed, it is essential that the phenotype of cells intended for recapitulation is fully elucidated. Cells of the adult human NP have routinely been likened to articular chondrocyte (AC) cells, with regards to both phenotype and morphology^6^. However, clear distinctions in the ECM produced by AC and NP cells have been demonstrated^7^, which has implications for the hydration state and stiffness of the tissue. This highlights the importance of accurate profiling of the NP cell phenotype and a number of microarray studies have been conducted in recent years in different species with a view to identifying a panel of marker genes distinguishing NP cells from other cell types, predominantly AC cells^8–12^. Our studies using both human and bovine NP and AC cells have identified a number of differentially expressed genes^9–11^, including forkhead box F1 (FOXF1), paired box 1 (PAX1), carbonic anhydrase 12 (CA12) and the keratins (KRT) 8, 18 and 19. These studies, along with those by others^11, 13^ have led to a consensus paper detailing a potential panel of human NP marker genes^14^. Importantly, however, studies to detail the NP phenotype at protein level are limited^15^ and thus further validation of newly identified NP markers at the protein level needs to be conducted. Crucially, localising the expression of NP marker proteins will allow for the elucidation of whether all, or only a subset of cells express these proteins.

One of the most interesting findings of the previous microarray investigations was the expression of previously described notochordal (NC) cell markers in cells of the adult human NP. KRT8, KRT18, KRT19, and brachyury (T) are expressed in the developing notochord, which is considered to be the developmental origin of the mature NP^16–18^. When compared to AC and AF cells, these genes were highly expressed in NP cells^8–12^. Furthermore, isolation of separate bovine NP and NC cell populations by size filtration with subsequent analysis of cellular gene expression identified similarities between the two cell types^10^. This is important as it is suggestive of a common ontogeny between NP and NC cells, or may be indicative of a subset of NP cells within the adult human NP that are phenotypically NC cell-like. Furthermore, analysis of cells isolated from non-degenerate human NP tissue and subsequently immortalised revealed NP cellular subpopulations at differing stages of maturation^19^, inferring that defining the NP cell phenotype requires an understanding of the changes in marker expression in development, ageing and degeneration.

The controversy regarding the phenotype and ontogeny of adult human NP cells described above highlights many unanswered questions, particularly regarding the heterogeneity of the adult NP cell population. As such we hypothesise that at least a proportion of cells present in the adult human NP are notochordally derived and that these cells persist irrespective of age or degeneration. Thus the aims of this investigation were: firstly to validate our previously described novel NP marker genes^9, 10^ in a large cohort of adult human specimens, and correlate levels of gene expression with age and severity of tissue degeneration; and secondly, to analyse expression of novel NP and NC cell marker proteins in cells of the adult human NP in order to ascertain whether expression is noted in all cells or a subset of NP cells, also correlating this expression to age and degenerative score.

## Results

### Novel NP and notochordal marker gene expression is maintained through age and degeneration

**Figure 1:**
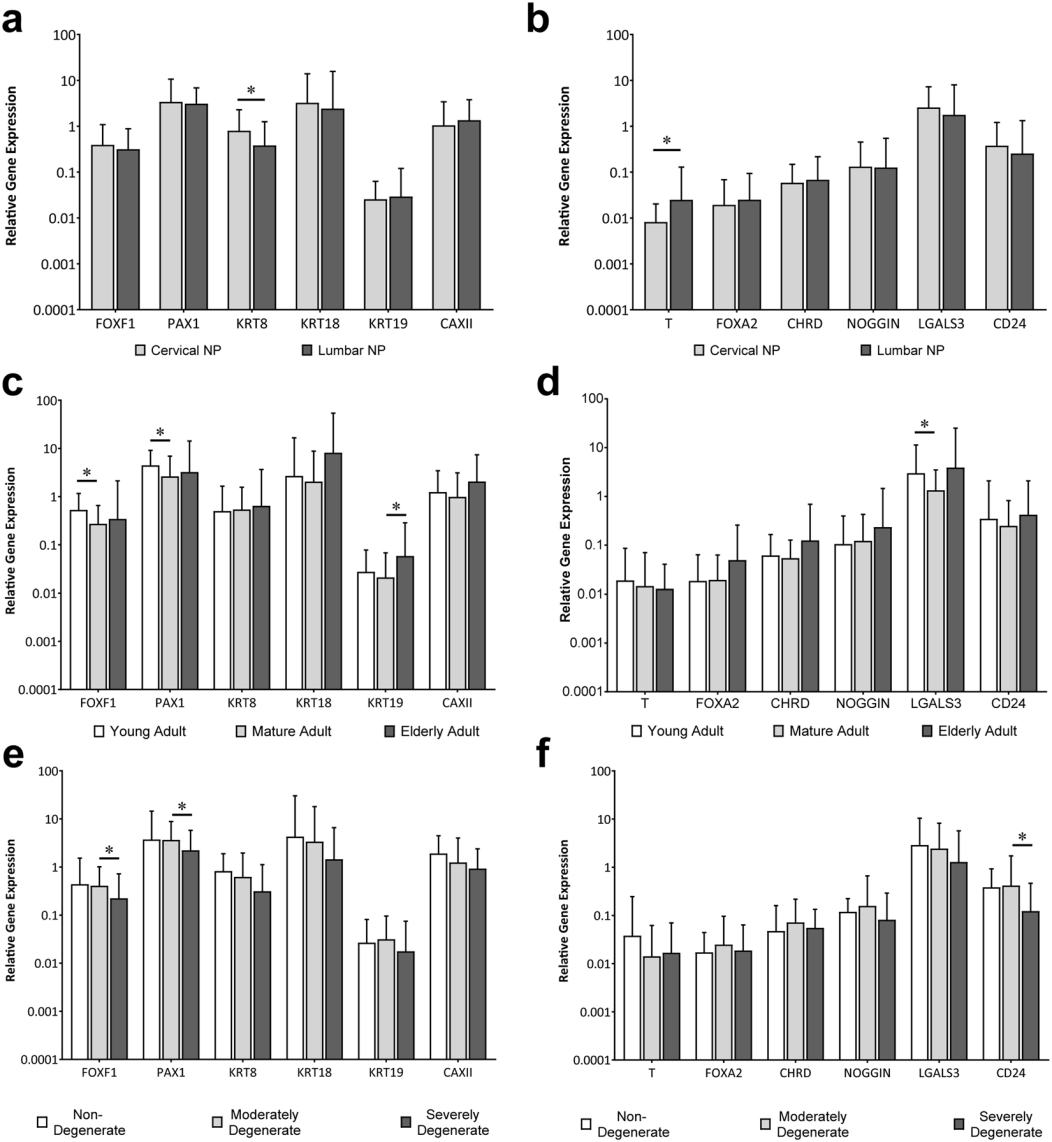
Novel NP and notochordal cell marker gene expression in adult human NP cells. Gene expression was compared between NP cells from (**a**) cervical (n = 27) and (**b**) lumbar (n = 33) discs. Expression was also stratified by (**c** and **d**) patient age (20–39 years n = 20; 40–59 years n = 30; 60+ years n = 10), and (**e** and **f**) by histological grade of degeneration (grade 0–4 n = 5; grade 5–7 n = 40; grade 8–12 n = 15). Gene expression was normalised to that of reference genes MRPL19 and EIF2B1. Values represent the mean ± SD. Statistical significance determined by Mann Whitney-U, *P < 0.05.

The gene expression levels of novel NP cell and notochordal cell marker genes were first assessed in NP cells of the IVD sample cohort. Gene expression analysis identified expression of all genes analysed in all samples. When comparing expression between NP cells from cervical and lumbar discs, only KRT8 and T demonstrated a significant difference, with KRT8 being higher in cervical NP and T being higher in lumbar NP (Fig. 1a and b). No significant regional variation was identified for any other NP or notochordal marker gene and thus subsequent analysis was performed on the entire cohort of disc samples.

Samples were then stratified by patient age, into categories of ‘young adult’ (20–39 years), ‘mature adult’ (40–59 years) and ‘elderly adult’ (60+ years) (Fig. 1c and d). Analysis demonstrated a significantly lower gene expression for both FOXF1 and PAX1 in mature adult as compared to young adult specimens, whilst KRT19 expression was higher in elderly adult versus mature adult samples. Expression levels of KRT8, KRT18 and CA12 did not vary significantly according to patient age. Gene expression levels of notochordal cell markers T, FOXA2, chordin (CHRD), noggin (NOG) and CD24 also did not differ across the various age categories, whilst LGALS3 expression was significantly lower in mature adult as compared to young adult NP samples.

When the gene expression levels of novel NP cell markers KRT8, KRT18, KRT19 and CA12 and notochordal markers T, FOXA2, CHRD, NOG and LGALS3 were analysed according to histological degenerative score, there was no significant variation in expression noted between non-degenerate, moderately degenerate and severely degenerate specimens (Fig. 1e and f). However, the NP markers FOXF1 and PAX1 and the notochordal marker CD24 all demonstrated a significant decrease in expression from moderately to severely degenerate NP.

Thus, at the transcript level, both novel NP and notochordal cell markers were consistently expressed within the NP cell population, irrespective of age or grade of degeneration.

### Differential expression of novel NP and notochordal marker protein expression in adult human IVD cells

**Figure 2:**
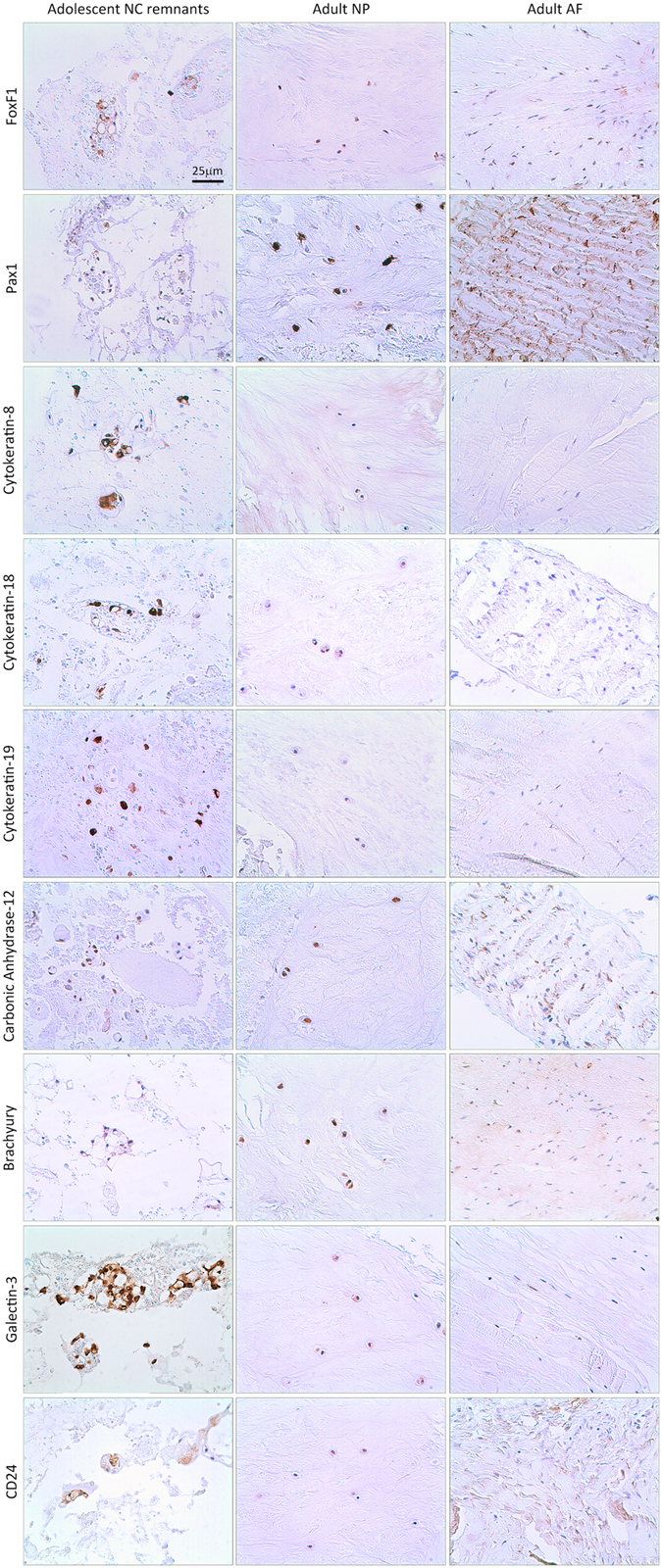
Immunolocalisation of novel NP cell and notochordal marker protein expression in cells of the IVD. Immunohistochemical staining for FoxF1, Pax-1, keratin-8, keratin-18, keratin-19, carbonic anhydrase-12, brachyury, galectin-3 and CD24 was assessed in adolescent human NP tissue, adult human NP tissue and adult human AF tissue. All images acquired at the same magnification and the scale bar = 25 μm.

Immunohistochemical staining of adult human IVD tissue revealed sub-populations of cells positive for the NP markers FoxF1, Pax-1, keratin-8, keratin-18 and carbonic anhydrase-12, and notochordal cell markers brachyury, galectin-3 and CD24 (Fig. 2) with positivity identified within both single small round cells and cells within clusters. Given the consistently low levels of gene expression across all samples of the notochordal markers FOXA2, CHRD and NOG, these proteins were not investigated using immunohistochemistry.

With the exception of keratin-19, immunopositivity was observed for all proteins analysed within all cohort specimens, although these were not ubiquitously expressed by all NP cells within the tissue. Interestingly, expression of all NP and NC markers was detected in notochordal remnant cells within the adolescent IVD tissue specimen (Fig. 2). These cells were localised in small clusters within the tissue and were identified as NC remnants by their large, round cell morphology which was distinct from that of mature NP cells. Keratin-19 staining demonstrated no immunopositivity in adult NP (Fig. 2), although staining was noted in NC remnant cells of adolescent IVD tissue. Additionally, staining was observed for all NP and NC cell markers in a proportion of AF cells within adult human IVD tissue where the AF region was evident (N = 21), with the exception of keratin-8, which could not be detected in the AF.

**Figure 3:**
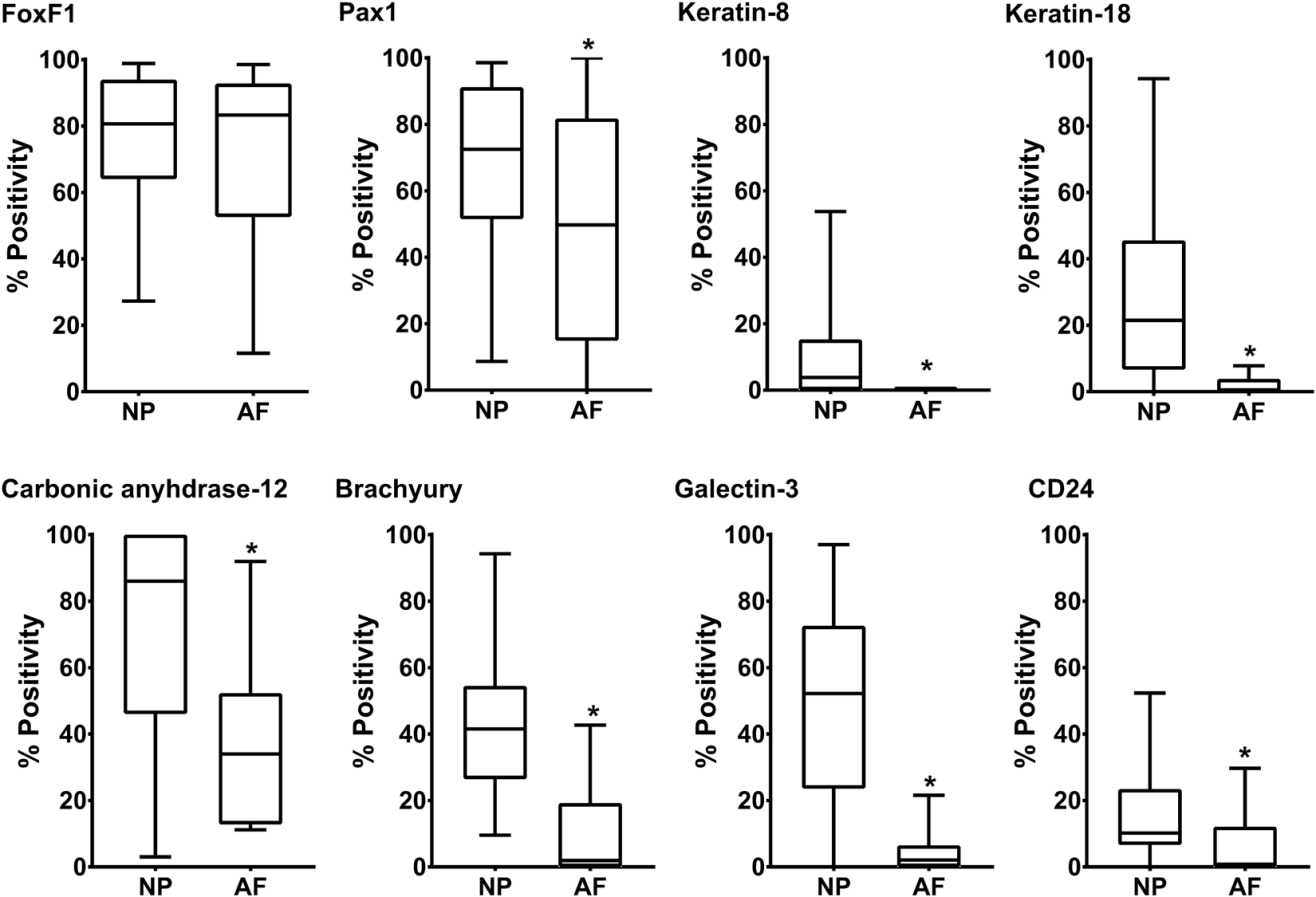
Percentage immunopositivity for NP and notochordal cell marker proteins FoxF1, Pax-1, keratin-8, keratin-18, carbonic anhydrase-12, brachyury, galectin-3 and CD24 in adult human NP and AF cells. Graphs represent median, with interquartile range and min/max values. Statistical significance determined by Mann Whitney-U, *P < 0.05.

The mean percentage immunopositivity across the cohort was quantified for all proteins analysed, irrespective of patient age or grade of degeneration, and percentage immunopositivity compared between NP and AF cells (Fig. 3). FoxF1 demonstrated high percentage positivity in both NP (84%) and AF (83%) cells, with no significant difference identified. All other markers demonstrated positivity in a significantly higher proportion of NP cells compared to AF cells (P ≤ 0.001 unless stated). Of note, both Pax-1 and carbonic anhydrase-12 demonstrated relatively high proportions of immunopositive cells in both NP and AF (71% vs. 50%, P = 0.04 for Pax-1; and 88% vs. 33% for carbonic anhydrase-12). The remaining markers demonstrated less than 50% cell immunopositivity in NP cells and less than 2% immunopositivity in AF cells, with keratin-8 being undetectable in AF cells from any sample.

### Sub-populations of NP cells immunopositive for novel NP and notochordal markers are maintained through ageing and degeneration

**Figure 4:**
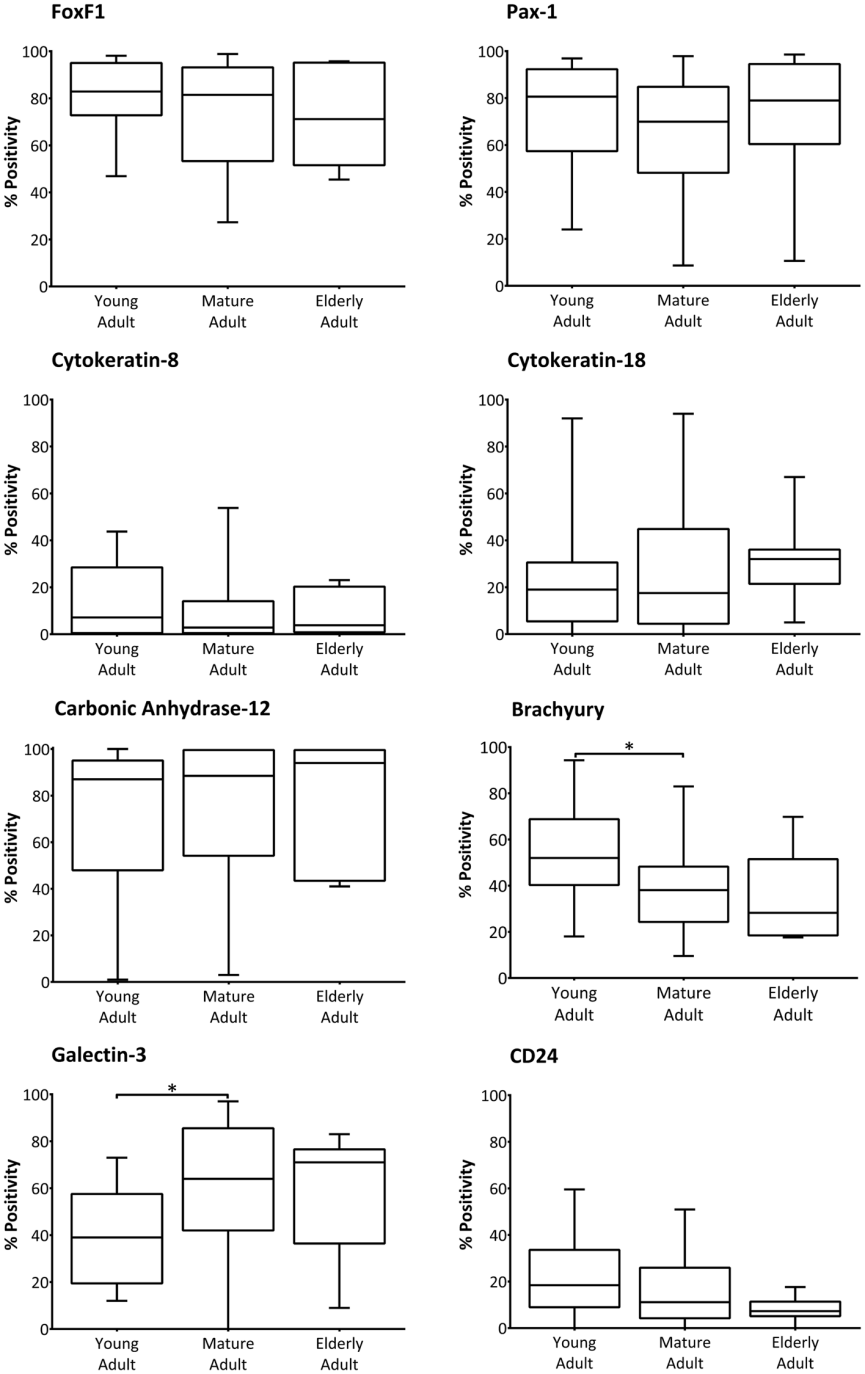
Immunopositivity for NP and NC cell marker proteins: variation with patient age. The percentage of adult human NP cells that stained positively for FoxF1, Pax-1, keratin-8, keratin-18, carbonic anhydrase-12, brachyury, galectin-3 and CD24 was assessed according to patient age (young adult 20–39 years, n = 20; mature adult 40–59 years, n = 28; elderly adult 60+ years, n = 7). Graphs represent median, with interquartile range and min/max values. Statistical significance determined by Mann Whitney-U, *P = <0.05.

Percentage cell positivity of marker proteins within the NP was assessed in different age groups and histological degenerative score. No significant variation was demonstrated in the cell positivity for any novel NP cell markers in the different age cohorts, or for the notochordal cell marker CD24 (Fig. 4). However, the notochordal cell marker protein brachyury demonstrated significantly lower percentage positivity in mature adult as compared to young specimens, whilst galectin-3 immunopositivity was higher in mature adult samples when compared to young adult.

**Figure 5:**
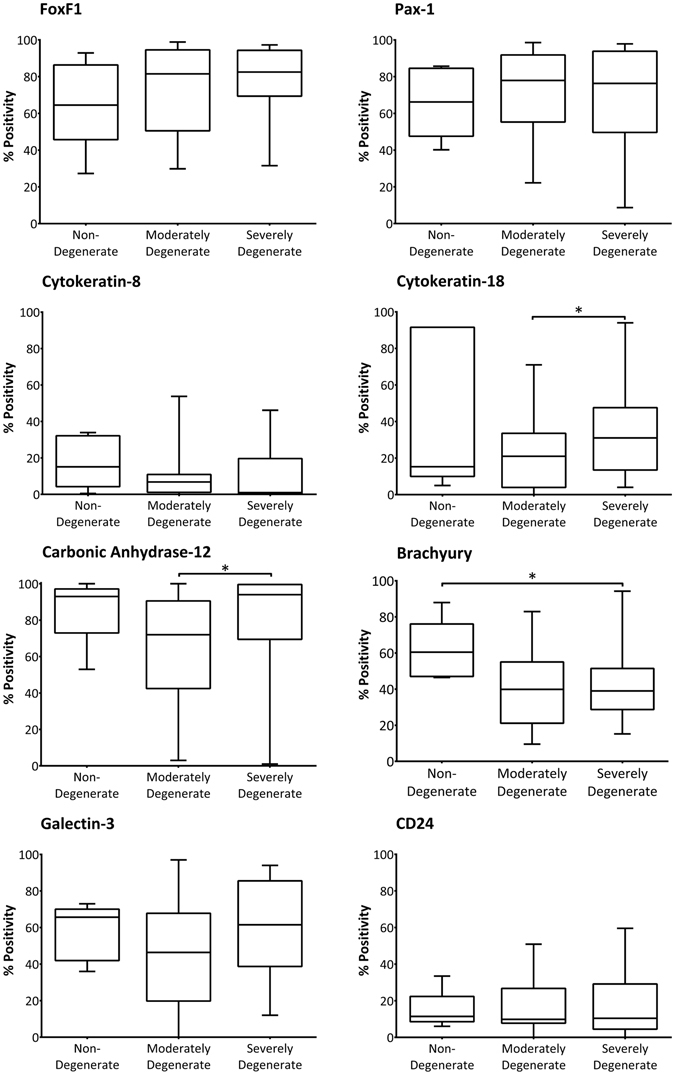
Immunopositivity for NP and NC cell marker proteins: variation with grade of degeneration. The percentage of adult human NP cells that stained positively for FoxF1, Pax-1, keratin-8, keratin-18, carbonic anhydrase-12, brachyury, galectin-3 and CD24 was assessed according to histological grade of tissue degeneration (non-degenerate grades 0–4, n = 6; moderately degenerate grades 5–7, n = 27; severely degenerate grades 8–12, n = 23). Graphs represent median, with interquartile range and min/max values. Statistical significance determined by Mann Whitney-U, *P < 0.05.

When percentage cell positivity of the novel NP and notochordal cell markers were analysed according to histological degenerative grade, there was generally no variation in the percentage of immunopositive cells (Fig. 5). The percentage of cells immunopositive for FoxF1, Pax-1, keratin-8, galectin-3 and CD24 did not vary significantly between non-degenerate, moderately degenerate or severely degenerate specimens. The proportion of NP cells expressing keratin-18 and carbonic anhydrase-12 was significantly higher in severely degenerate tissue samples as compared to moderately degenerate specimens (P = 0.047 and 0.018 respectively), whilst the percentage of cells immunopositive for brachyury was significantly lower in severely degenerate tissues compared to non-degenerate (P = 0.039).

### Multi-parametric flow cytometry analysis confirms presence of NC-like cells within the adult human NP

**Figure 6:**
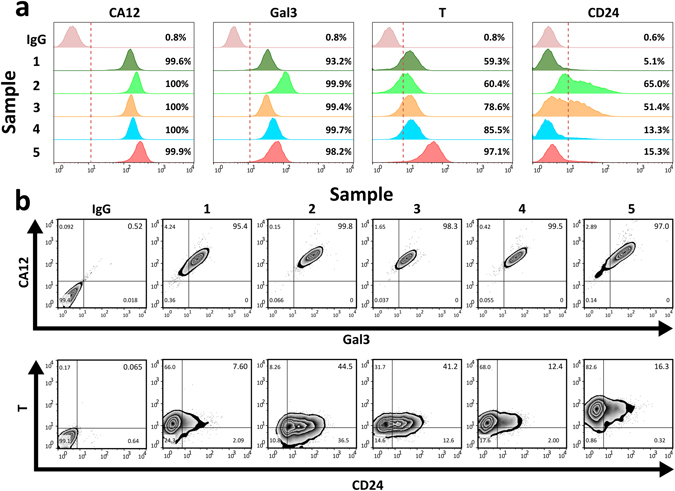
Multiparametric flow cytometry reveals NP cell subpopulations with a notochordal phenotype. (**a**) Single channel flow cytometry for carbonic anhydrase-12 (CA12), galectin-3 (Gal3), brachyury (T) and CD24 and isotype (IgG) controls in five freshly isolated adult human NP cell samples. Percentage positivity for each marker in each sample is provided. (**b**) Multiparametric flow cytometry for expression of CA12, Gal3, T and CD24 in the same five adult human NP samples. Viable single cells were gated for CA12 and Gal3 dual positivity (top row). CA12^+^Gal3^+^ NP cells were then gated for T and CD24 positivity (bottom row) to provide a percentage of CA12^+^Gal3^+^T^+^CD24^+^ cells expressing a NC-like phenotype.

Following immunohistochemical analysis, the expression of four markers was assessed by flow cytometry (Fig. 6). Analysis of freshly-isolated cells from five surgical NP samples demonstrated that an average of 99.9% (SD ± 0.2%) of adult NP cells expressed carbonic anhydrase-12, while an average 98.1% (SD ± 2.8%) were positive for galectin-3. However, immunopositivity of brachyury and CD24 was found to be far more variable, with brachyury positivity ranging from 59% to 97% (mean 76.2% (SD ± 16.3%)), and CD24 from 5% to 65% (mean 30.0% (SD ± 26.4%)) (Fig. 6a). Co-expression analysis revealed that while 98% (SD ± 1.83%) of adult human NP cells were CA12^+^Gal3^+^, only 24.4% (SD ± 17.2%) of cells were CA12^+^Gal3^+^T^+^CD24^+^ (Fig. 6b).

## Discussion

Recent years have seen the publication of panels of novel IVD cell markers that may aid in the demarcation of NP cells from other cell types^8–12^, but few have validated their mRNA expression in a large cohort of specimens encompassing a range of spinal levels, patient ages and degenerative scores or investigated the protein expression of these newly identified markers. The NP markers used here were identified from studies on a number of species, including rat^12^, canine^8^, bovine^10^ and human^9, 11^, and are part of a panel of agreed NP phenotypic markers^14^.

In this study we have demonstrated in a surgical cohort that both novel NP and NC markers are expressed at similar levels between NP specimens of cervical and lumbar discs, and that novel NP and NC marker gene expression is largely maintained in ageing and degeneration. Furthermore with the exception of KRT8 and T, NP marker gene expression was comparable between cervical and lumbar NP cells, suggesting that distinctions in tissue matrix composition, load and anatomy^20–22^ do not influence NP cell phenotype. Analysis of protein expression highlighted non-ubiquitous expression of the novel NP markers within the NP and the expression of NC markers within a smaller proportion of NP cells, with little significant variation in the proportion of NP cell immunopositivity noted in ageing and degeneration.

Gene expression analysis demonstrated that expression of all novel NP and notochordal cell marker genes could be identified in all samples within this large human IVD cohort, confirming findings from our previous study^9^ and that by Thorpe and colleagues^15^. Furthermore levels of expression of these genes generally did not vary with either age or degeneration, with only minor changes in a small subset of genes identified. The sustained expression of both CHRD and NOG genes by adult human NP cells confirms previous findings which demonstrated their expression by both human NP and AF cells and may highlight a role for these molecules as BMP antagonists in preventing ossification of the disc^23^. However, the previously identified expression by AF cells, combined with the low levels of expression identified here meant these genes were not studied further.

Importantly this study demonstrates expression of notochordal marker genes in the adult human NP, which are maintained throughout ageing and degeneration, suggesting a notochordal origin for at least a proportion of cells present within the adult disc. However, one of the limitations of gene transcript analysis is the global view of expression that is obtained, meaning it is not possible to determine whether expression is ubiquitous or specific to subsets of cells. Here, confirmation of expression at protein level and localisation of immunopositive cells within adult human IVD tissue has highlighted that expression of these novel NP and notochordal markers is in fact heterogeneous.

In the cohort of samples used in this study 22 contained AF tissue. When comparing NP and AF regions all novel NP marker proteins, except FoxF1, demonstrated significantly higher numbers of immunopositive cells in the NP compared to the AF. Interestingly we first identified FoxF1 as an IVD-specific, rather than NP-specific cell marker^9^ and thus the relatively high numbers of immunopositive NP and AF cells is unsurprising. The findings also confirm previous studies demonstrating expression of FoxF1 by AF cells at both the gene and protein level^10, 15, 24^. While significantly lower than NP, a relatively high proportion of AF cells displayed positivity for Pax-1 and carbonic anhydrase-12, supporting previous evidence of expression in human AF cells^15, 24^. Conversely keratin-8 and -19 expression was absent from AF cells, with <2% of AF cells demonstrating positivity for keratin-18, highlighting the specificity of keratins as NP-specific markers.

Immunolocalisation analysis of our novel NP markers within the adult human NP demonstrated no significant change in the number of FoxF1 or Pax1 immunopositive cells with ageing or degeneration. However, there was a significant increase in carbonic anhydrase-12 immunopositivity with degeneration, in agreement with previous work where the most intense staining was found to be in the most degenerate tissues^11^.

This is the first investigation into NC marker expression within the adult human NP in a large sample cohort and one of the most pertinent findings of this study was the expression of notochordal marker genes and persistence of a subset of cells which express notochordal markers in adult degenerate NP tissues. Decreases in keratin-8, keratin-18 and keratin-19 protein expression have previously been correlated with ageing in human discs^25^; although no keratin-19 expression was detected in the present study. The previous investigation used a pan-keratin antibody for assessment of keratin-19 staining, and given the results presented here, likely represents expression of keratin-8 rather than -19, which would support data from another study where keratin-19 protein becomes undetectable shortly after adolescence^26^.

The literature regarding the notochordal marker brachyury protein expression in adult NP is conflicting. Immunonegativity has been reported in adult human NP cells^18^, whilst conversely Western blot analysis has revealed expression within adult human cells^27^. Significantly our data demonstrates immunopositivity for brachyury in a large cohort of adult human NP cells, which is maintained with ageing and degeneration, although both age- and degeneration-related decreases were seen. We have also shown the presence of galectin-3 and a small number of CD24 positive cells within the adult human NP, indicative of an NC-like cell population existing within the mature NP. Galectin-3 and CD24 have previously been localised to rodent and human NP cells^25, 28–31^. While galectin-3 has previously been reported as lost with ageing^25^ and CD24 not correlated to age or degenerative score in human tissues^28^, our data suggest that expression of an NC-like phenotype is maintained in a small number of adult NP cells in ageing and degeneration of the adult human IVD.

To confirm presence of cells with an NC-like phenotype within the adult human NP, we analysed co-expression of the ubiquitously expressed NP marker carbonic anhydrase-12, with the NC markers galectin-3, brachyury and CD24. Flow cytometry analysis confirmed high cell positivity for carbonic anhydrase-12 and galectin-3, with 98% of adult human NP cells being CA12^+^Gal3^+^, supporting our immunolocalisation studies. Further analysis also revealed a smaller population of CA12^+^Gal3^+^T^+^CD24^+^ adult human NP cells from surgically isolated samples across a range of patient ages, supporting our growing evidence that at least a sub-population of adult human NP cells are notochordally derived and retain notochordal marker expression.

The persistence of cells with an NC-like phenotype, but with an NP-like morphology, within the adult human NP is still a matter of debate, with many recent studies reporting the presence of cell sub-populations within the NP. These include the possible identification of MSC-like cell populations within the adult IVD^32, 33^, and TIE2^+^GD2^+^ NP cells capable of multi-lineage differentiation^34^. Taken together, these studies indicate that a proportion of IVD cells possess a progenitor-like phenotype, which is likely diminished or rendered ineffective with progressing age or degeneration. However, none of these studies investigated the possibility of an NC cell sub-population within adult NP tissue. In humans, morphologically-distinct NC cells disappear from the NP with ageing^35^. It has been suggested that NC cells confer some protective effect over NP cells through maintenance of IVD integrity and tissue ECM^36–38^ and through protection from degradation and apoptosis^39^. Thus, our NC-marker^+^ cell sub-population may persist within the tissue as an endogenous attempt at tissue repair, protecting the NP from the effects of early tissue degeneration. However this is purely speculative at present and requires further investigation by isolation of distinct cell populations. Importantly, the identification of NC marker expression within adult human NP cells supports the notion that at least a proportion of cells of the adult human NP are notochordally-derived^40^, rather than as a result cellular infiltration from surrounding tissues^18^. Further analysis of these putative NC-like cells will help evolve our understanding of IVD homeostasis and repair, as well as provide a source of cells for future therapeutic interventions.

## Materials and Methods

### Tissue acquisition and processing

Adult human IVD specimens (Supplementary Table 1) were obtained with informed written consent from patients undergoing disc surgery for treatment of IVD degeneration with National Research Ethics Service committee approval (08/H1010/36), and all experimental protocols were performed in accordance with National Research Ethics Service and University of Manchester guidelines and regulations. Adolescent IVD tissue was obtained from the cadaver of an 18 year old, with informed written consent from the donor’s relatives and National Research Ethics Service committee approval. The tissue was used for immunohistochemistry studies due to the presence of notochordal remnant cells within the NP, but was not included in gene expression analysis or statistical comparisons.

Prior to digestion, a portion of tissue containing both NP and AF was washed in PBS, fixed in 4% paraformaldehyde, processed to paraffin wax and 5μm sections cut. Using H&E stained sections; specimens were graded histologically by a histopathologist (AJF) for level of IVD degeneration as previously described^6^. When samples were analysed according to degenerative score, three classifications were utilised: non-degenerate (grade 0–4); moderately degenerate (grade 5–7); and severely degenerate (grade 8–12).

NP tissue from each sample was macroscopically dissected from AF, and macerated prior to enzymatic digestion in a solution of 0.1% (w/v) type II collagenase (Thermo Fisher; 17101-015) and 0.1% (w/v) hyaluronidase (Sigma Aldrich; H3506) in 5 ml per gram of tissue serum-free DMEM overnight at 37 °C with agitation. Isolated cells were cultured in DMEM supplemented with 10% (v/v) FBS, 1 mM sodium pyruvate, 10,000 U/ml penicillin, 10 mg/ml streptomycin, 25 μg/ml amphotericin B and 1 mM ascorbate under standard conditions for approximately 10 days (37 °C, 21% O_2_, 5% CO_2_). Once cells had reached 80% confluency, cells were lysed using Trizol® reagent.

### Gene expression analysis

RNA extraction and cDNA synthesis was performed according to previously published methodology^9, 10^. Intron-spanning qPCR assays (Supplementary Table 2) were designed against human gene sequences and qPCR reactions conducted on a StepOnePlus system (Applied Biosystems), using FAM-BHQ1 probes and corresponding primers (Sigma Aldrich). A total of 10 ng cDNA was added to each reaction in addition to 5μl LuminoCt qPCR ReadyMix (Sigma Aldrich), 1μl forward primer, 1μl reverse primer, 0.5μl probe and 0.5μl molecular biology grade water. All primer concentrations were optimised empirically, and all primers were used at 900 nM, except EIF2B1 and T, which were used at a 300 nM final concentration. Each probe was used at a final concentration of 250 nM. cDNA generated in-house from Total Human RNA or Foetal RNA (Clontech) was used as a positive control. Gene expression levels for novel NP and notochordal marker genes were normalised against pre-validated reference genes MRPL19 and EIF2B1. qPCR data was analysed according to the 2^−ΔCt^ method^9, 10, 41^ and statistical analysis was performed using GraphPad InStat and GraphPad Prism software and Mann Whitney-U tests where significance was defined as p < 0.05.

### Immunohistochemical analysis

Immunohistochemical staining was conducted on 5 µm paraffin sections of IVD tissue (Supplementary Table 1). Antigen retrieval methods were as follows: 0.25% (w/v) pepsin incubation for Brachyury and FoxF1 staining; pressure cooker treatment with citrate buffer pH6.0 for Carbonic anhydrase-12 and Pax-1 staining; steamer treatment with citrate buffer pH6.0 for CD24 staining; 0.25% (w/v) pepsin incubation followed by 0.1% (w/v) pronase incubation for keratin-8 and Galectin-3 staining; and 0.25% (w/v) pepsin incubation followed by steamer treatment with Tris EDTA pH9.0 for keratin-18 and -19 staining. Sections were incubated overnight at 4 °C with primary antibody (brachyury, Abcam ab57480, 1:10; carbonic anhydrase-12, Sigma HPA008773, 1:50; CD24, Abcam ab31622, 1:50; keratin-8, Zytomed 603-2156, 1:10; keratin-18, Dako M7010, 1:20; keratin-19, Dako M0888, 1:10; FoxF1, Santa Cruz sc-47591, 1:35; galectin-3, Santa Cruz sc-20157, 1:20; and Pax-1, Abcam ab114037, 1:600) diluted in 25% serum in tris-buffered saline (TBS) pH7.6, whilst negative controls were incubated with the appropriate isotype control also diluted in 25% serum in TBS pH7.6. Goat anti-mouse, goat anti-rabbit or donkey anti-goat secondary antibodies (Abcam) were diluted in 25% serum in TBS pH7.6 at a working concentration of 0.3μg/ml and applied to the sections for 30 minutes. Staining was amplified using Avidin-Biotin Complex (ABC) reagent (Vector Labs) for 30 minutes and a 3,3′-diaminobenzidine tetrahydrochloride (DAB) disclosure system utilised (Sigma Aldrich). Sections were counterstained with Mayer’s haematoxylin and staining was assessed by light microscopy, and images acquired using an InfinityX camera with DeltaPix software. Cell positivity was quantified by counting all NP and AF cells within an IVD specimen and identifying the number of cells staining positively for the protein of interest. For statistical analysis, Mann Whitney-U tests were performed using GraphPad Prism software.

### Flow Cytometry Analysis

Cells were isolated from the NP of five donors undergoing IVD surgery for low back pain (18–50 years; mean 35.4 years) as previously described^9^. For each sample 1 × 10^6^ freshly isolated cells were stained using antibodies for CD24-Pacific blue (Exbio Antibodies, PB-503-T100; 2.5 ug/ul), brachury-APC (R&D Systems, IC20851A; 150 ug/ul), galectin-3-Alexa Fluor488 (R&D Systems, IC1154G; 200 ug/ul) or carbonic anhydrase-12-PE (Bioss USA, bs-6025R-PE; 100 ng/ul); a duplicate sample was stained using the relevant conjugated IgG controls. Samples were analysed using a CyanADP flow cytometer and presented using FlowJo software.

## Electronic supplementary material

Supplementary information
