# Supplementary material for: Notochordal and nucleus pulposus marker expression is maintained by sub-populations of adult human nucleus pulposus cells through aging and degeneration
Source: Sci Rep. 2017 May 4;7:1501. doi: 10.1038/s41598-017-01567-w (PMC5431421; doi:10.1038/s41598-017-01567-w)
Supplement: Supplementary file 1 — Supplementary information [file 41598_2017_1567_MOESM1_ESM.pdf]

Title: Notochordal and nucleus pulposus marker expression is maintained by sub-populations of adult human nucleus pulposus cells through aging and degeneration

Authors: Stephen M. Richardson[1+*] (s.richardson@manchester.ac.uk)

Francesca E. Ludwinski[1+] (francesca.e.ludwinski@kcl.ac.uk)

Kanna K. Gnanalingham[2] (Kanna.Gnanalingham@srft.nhs.uk)

Ross Atkinson[2] (ross.atkinson@manchester.ac.uk)

Anthony J. Freemont FRCPath[1,3] (tony.freemont@manchester.ac.uk)

Judith A. Hoyland[1,3] (judith.a.hoyland@manchester.ac.uk)

Supplementary information

Supplementary table 1: Details of the samples used for qPCR and/or immunohistochemistry (IHC), or flow cytometry, including disc level, patient age, gender and grade of degeneration. Numbers in the flow cytometry column relate to samples numbers presented in figure 6.

Disc level	Age	Gender	Grade	qPCR	IHC IVD Tissue		Flow cytometry
					NP	AF	
C3/4	45	M	3	•	•		
C3/4	72	F	6	•			
C4/5	48	M	6	•	•		
C4/5	50	F	8	•	•		
C4/5	55	F	6	•	•		
C4/5	55	F	9	•	•		
C4/5	72	F	7	•			
C5/6	18	M	6				1
C5/6	34	M	6	•	•	•	
C5/6	38	F	9	•	•		
C5/6	39	M	4	•	•	•	
C5/6	43	F	4	•	•	•	
C5/6	46	F	5	•	•	•	
C5/6	47	M	6	•	•	•	
C5/6	48	F	4	•			
C5/6	48	M	5	•	•		
C5/6	48	M	10	•	•	•	
C5/6	50	F	6	•	•		
C5/6	50	M	10				5
C5/6	59	M	7	•	•		
C5/6	60	F	7	•	•	•	
C5/6	63	M	6	•	•		
C6/7	33	M	9	•	•		
C6/7	34	M	5	•	•	•	
C6/7	34	F	5	•	•	•	
C6/7	43	M	5	•	•		
C6/7	45	F	5	•	•		
C6/7	46	F	9	•	•	•	
C6/7	49	M	0		•	•	
C6/7	50	F	8	•	•		
C6/7	50	F	9				2
L3/4	42	M	7	•	•		
L3/4	51	M	10	•	•	•	
L3/4	57	M	10	•	•		
L4/5	23	M	6		•		
L4/5	25	M	6	•	•	•	
L4/5	25	M	7	•	•	•	
L4/5	27	M	12				4

L4/5	31	M	7	•	•		
L4/5	31	M	9	•	•		
L4/5	36	F	9	•	•	•	
L4/5	41	M	8	•			
L4/5	42	M	7	•	•		
L4/5	44	M	8	•	•	•	
L4/5	44	F	8	•	•		
L4/5	48	F	5	•	•	•	
L4/5	58	M	10	•	•		
L4/5	65	F	6	•	•		
L4/5	68	F	7	•	•		
L4/5	75	F	9	•	•	•	
L4/5	80	F	8	•	•		
L5/S1	22	M	4	•	•		
L5/S1	26	M	6	•	•		
L5/S1	26	F	6	•	•		
L5/S1	27	F	8	•	•		
L5/S1	32	F	6				3
L5/S1	35	F	7	•	•	•	
L5/S1	37	F	7	•	•	•	
L5/S1	37	M	8	•	•		
L5/S1	37	F	9	•	•		
L5/S1	38	F	11	•	•		
L5/S1	40	F	10	•	•		
L5/S1	41	F	7	•	•	•	
L5/S1	46	F	7	•	•		
L5/S1	46	M	8	•	•		
L5/S1	60	F	8	•			
L5/S1	60	M	10	•	•		

Supplementary table 2: qPCR Assays for NP and notochordal marker genes.

Gene Name	Accession Number	Forward Primer Sequence 5'-3'	Reverse Primer Sequence 5'-3'	Probe Sequence 5'-3'
MRPL19	NM_014763	CACCGCCCCGTGGAA	TCCCCTTCGAGGAATGAATTC	AACGCAGGTTCTTGAGTC
EIF2B1	NM_001414	TCATCAAAGATGGAGCGACAATA	CCAGGACTCTCAGGACCACTCT	TGACTCACGCCTACTC
FOXF1	NM_001451	GCCGTATCTGCACCAGAACA	CGTTGAAAGAGAAGACAAACTCCTT	CTGCAAGGCATCCCG
PAX-1	NM_006192	ACCCCCGCAGTGAATGG	GGCCGACTGAGTGTATTTAATGTCT	CTAGAGAAACCTGCCTTAGA
KRT8	NM_002273	TGACCGACGAGATCAACTTCCT	TGGACAGCACCACAGATGTGT	CAGCTATATGAAGAGGAGATC
KRT18	NM_199187	GCGAGGACTTTAATCTTGGTGATG	TGGTCTTTTGGATGGTTTGCA	CAGCAGCAACTCC
KRT19	NM_002276	GGTCATGGCCGAGCAGAA	TTCAGTCCGGCTGGTGAAC	CGGAAGGATGCTGAAG
CAXII	NM_001218	CGTGCTCCTGCTGGTGATCT	AGTCCACTTGGAACCGTTCACT	AAAGGAACAGCCTTCCAG
NOTO	NM_001134462	AGTTGGAGAAAGTGTTTG	CTGCTTCTGATACTTGAC	AGACTCTCACGTGGTTCTCTGT
FOXJ1	NM_001454	GAGCAACTTCTTCCAGAA	CCTCCTCCGAATAAGTATG	CTGCGCTCTGAGCCAGGCAC
FOXA2	NM_021784	AGCAGCTACTATGCAGAG	GCTCATGTACGTGTTCAT	AGGGCTACTCCTCCGTGAGC
T	NM_003181	TTCTCCAACCTATTCTGACAACTCA	ATTCCAAGGCTGGACCAATTG	TTTATCCATGCTGCAATC
CHRD	NM_003741	CCGCTTCTCTATCTCCTA	GAGTGAGTGTCACAAGTG	CTAAGGAGCCGCAGAGACAACC
NOGGIN	NM_005450	AGCTATAGAGTTCAATGTTAT	GCACTTCTTTCTCATTTAC	ATAGAGAACAAATGGAATGACTAATCA
LGALS3	NM_001177388	CAGACAATTTTTCGCTCCATGA	GGCCATCCTTGAGGGTTTG	CGTTATCTGGGTCTGGAAA
CD24	NM_0013230	GCTCCTACCCACGCAGATTTAT	CCTTGGTGGTGGCATTAGTTG	CCAGTGAAACAACAAC

Supplementary Figure 1

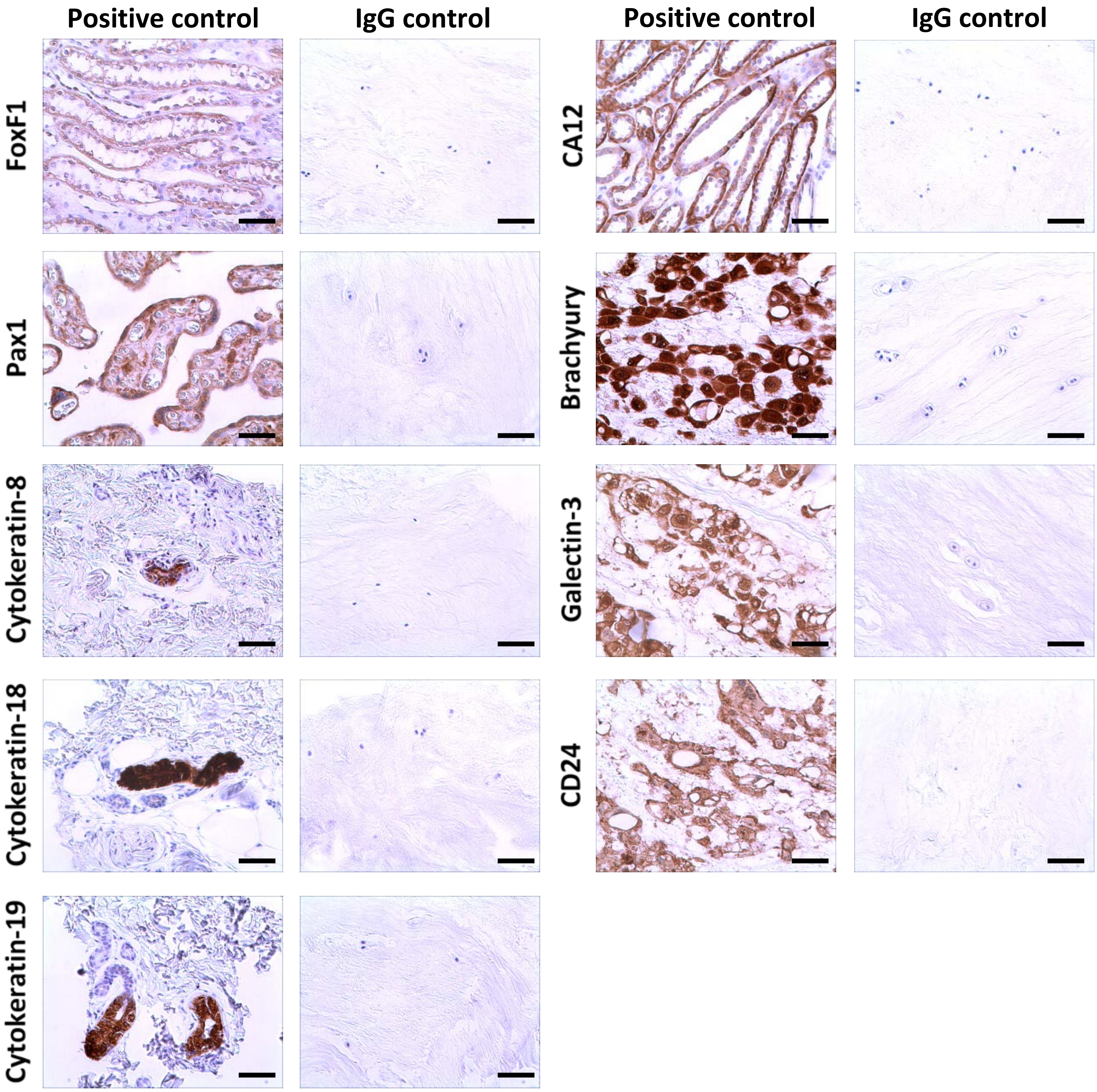

Supplementary figure 1: Positive and IgG controls for immunohistochemical staining of novel NP and notochordal cell marker proteins. IgG controls were performed on human IVD tissue at equivalent protein concentrations to primary antibodies. Positive control tissues were: human kidney for FoxF1 and carbonic anhydrase-12; human placenta for Pax1; human glandular epithelium for cytokeratins 8, 18 and 19; and human chordoma for brachyury, galectin-3 and CD24. Scale bar = 50µm.
